# 4-Methyl-1-(4-methylbenzylidene)thio­semicarbazide

**DOI:** 10.1107/S1600536810040663

**Published:** 2010-10-20

**Authors:** Yu-Feng Li

**Affiliations:** a Microscale Science Institute, Department of Chemistry and Chemical Engineering, Weifang University, Weifang 261061, People’s Republic of China

## Abstract

The title compound, C_10_H_13_N_3_S, prepared by the reaction of 4-methyl­benzaldehyde and 4-methyl­thio­semicarbazide, is approximately planar (r.m.s. deviation for the non-H atoms = 0.032 Å). Its conformation is stabilized by an intra­molecular N—H⋯N hydrogen bond, generating an *S*(5) ring. In the crystal, inversion dimers linked by pairs of N—H⋯S hydrogen bonds occur. Further weak N—H⋯S links connect the dimers into (100) sheets.

## Related literature

For related structures, see: Li & Jian (2010 ▶); Li *et al.* (2009 ▶).
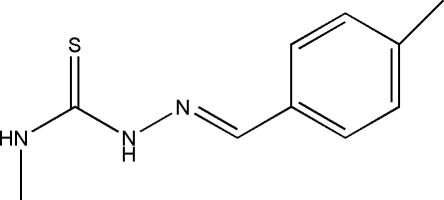


## Experimental

### 

#### Crystal data

C_10_H_13_N_3_S
*M* *_r_* = 207.29
Monoclinic, 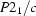

*a* = 9.1139 (18) Å
*b* = 13.689 (3) Å
*c* = 9.1195 (18) Å
β = 91.92 (3)°
*V* = 1137.1 (4) Å^3^
*Z* = 4
Mo *K*α radiation
μ = 0.25 mm^−1^
*T* = 293 K
0.25 × 0.23 × 0.20 mm

#### Data collection

Bruker SMART CCD diffractometer
10620 measured reflections
2585 independent reflections
1739 reflections with *I* > 2σ(*I*)
*R*_int_ = 0.030

#### Refinement

*R*[*F* ^2^ > 2σ(*F* ^2^)] = 0.044
*wR*(*F* ^2^) = 0.146
*S* = 1.14
2585 reflections
127 parameters
H-atom parameters constrained
Δρ_max_ = 0.28 e Å^−3^
Δρ_min_ = −0.19 e Å^−3^

### 

Data collection: *SMART* (Bruker, 1997 ▶); cell refinement: *SAINT* (Bruker, 1997 ▶); data reduction: *SAINT*; program(s) used to solve structure: *SHELXS97* (Sheldrick, 2008 ▶); program(s) used to refine structure: *SHELXL97* (Sheldrick, 2008 ▶); molecular graphics: *SHELXTL* (Sheldrick, 2008 ▶); software used to prepare material for publication: *SHELXTL*.

## Supplementary Material

Crystal structure: contains datablocks global, I. DOI: 10.1107/S1600536810040663/hb5666sup1.cif

Structure factors: contains datablocks I. DOI: 10.1107/S1600536810040663/hb5666Isup2.hkl

Additional supplementary materials:  crystallographic information; 3D view; checkCIF report

## Figures and Tables

**Table 1:** Hydrogen-bond geometry (Å, °)

*D*—H⋯*A*	*D*—H	H⋯*A*	*D*⋯*A*	*D*—H⋯*A*
N1—H1*A*⋯N3	0.86	2.27	2.644 (2)	107
N1—H1*A*⋯S1^i^	0.86	2.89	3.4869 (16)	128
N2—H2*A*⋯S1^ii^	0.86	2.57	3.4205 (18)	171

Symmetry codes: (i) 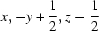; (ii) 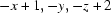.

**Fig. 1.**
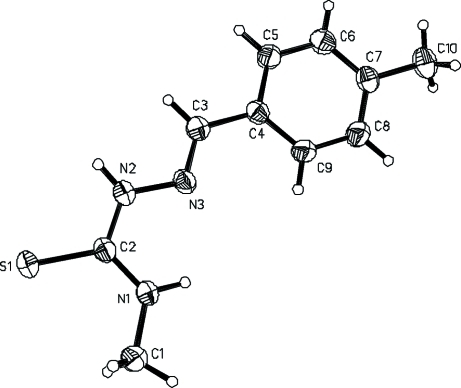
The structure of (I) showing 30% probability displacement ellipsoids.

## supplementary crystallographic information

### Experimental

A mixture of the 4-methylbenzaldehyde (0.10 mol) and 4-methylthiosemicarbazide (0.10 mol) was stirred in refluxing ethanol (10 mL) for 4 h to afford the title compound (0.078 mol, yield 78%). Colourless bars of (I) were obtained by recrystallization from ethanol at room temperature.

### Refinement

H atoms were fixed geometrically and allowed to ride on their attached atoms, with C—H distances = 0.93–0.97 Å; N—H = 0.86Å and with *U*_iso_(H) = 1.2*U*_eq_(C,*N*) or 1.5*U*_eq_(C_methyl_).

### Crystal data

**Table:**

C_10_H_13_N_3_S	*F*(000) = 440
*M**_r_* = 207.29	*D*_x_ = 1.211 Mg m^−^^3^
Monoclinic, *P*2_1_/*c*	Mo *K*α radiation, λ = 0.71073 Å
Hall symbol: -P 2ybc	Cell parameters from 1840 reflections
*a* = 9.1139 (18) Å	θ = 3.3–25.2°
*b* = 13.689 (3) Å	µ = 0.25 mm^−^^1^
*c* = 9.1195 (18) Å	*T* = 293 K
β = 91.92 (3)°	Bar, colorless
*V* = 1137.1 (4) Å^3^	0.25 × 0.23 × 0.20 mm
*Z* = 4	

### Data collection

**Table:**

Bruker SMART CCD diffractometer	1739 reflections with *I* > 2σ(*I*)
Radiation source: fine-focus sealed tube	*R*_int_ = 0.030
graphite	θ_max_ = 27.5°, θ_min_ = 3.5°
phi and ω scans	*h* = −11→11
10620 measured reflections	*k* = −16→17
2585 independent reflections	*l* = −11→11

### Refinement

**Table:**

Refinement on *F*^2^	Primary atom site location: structure-invariant direct methods
Least-squares matrix: full	Secondary atom site location: difference Fourier map
*R*[*F*^2^ > 2σ(*F*^2^)] = 0.044	Hydrogen site location: inferred from neighbouring sites
*wR*(*F*^2^) = 0.146	H-atom parameters constrained
*S* = 1.14	*w* = 1/[σ^2^(*F*_o_^2^) + (0.0782*P*)^2^ + 0.0376*P*] where *P* = (*F*_o_^2^ + 2*F*_c_^2^)/3
2585 reflections	(Δ/σ)_max_ = 0.001
127 parameters	Δρ_max_ = 0.28 e Å^−^^3^
0 restraints	Δρ_min_ = −0.19 e Å^−^^3^

### Special details

**Table:**

Geometry. All e.s.d.'s (except the e.s.d. in the dihedral angle between two l.s. planes) are estimated using the full covariance matrix. The cell e.s.d.'s are taken into account individually in the estimation of e.s.d.'s in distances, angles and torsion angles; correlations between e.s.d.'s in cell parameters are only used when they are defined by crystal symmetry. An approximate (isotropic) treatment of cell e.s.d.'s is used for estimating e.s.d.'s involving l.s. planes.
Refinement. Refinement of *F*^2^ against ALL reflections. The weighted *R*-factor *wR* and goodness of fit *S* are based on *F*^2^, conventional *R*-factors *R* are based on *F*, with *F* set to zero for negative *F*^2^. The threshold expression of *F*^2^ > σ(*F*^2^) is used only for calculating *R*-factors(gt) *etc*. and is not relevant to the choice of reflections for refinement. *R*-factors based on *F*^2^ are statistically about twice as large as those based on *F*, and *R*- factors based on ALL data will be even larger.

### Fractional atomic coordinates and isotropic or equivalent isotropic displacement parameters (Å^2^)

**Table:**

	*x*	*y*	*z*	*U*_iso_*/*U*_eq_	
S1	0.32325 (7)	0.10479 (4)	1.03876 (5)	0.0635 (2)	
N3	0.52576 (18)	0.10902 (11)	0.66835 (16)	0.0500 (4)	
N2	0.48287 (19)	0.08528 (11)	0.80716 (16)	0.0546 (4)	
H2A	0.5276	0.0396	0.8555	0.065*	
N1	0.30549 (18)	0.20245 (12)	0.78748 (16)	0.0577 (4)	
H1A	0.3375	0.2139	0.7015	0.069*	
C2	0.3712 (2)	0.13330 (14)	0.86754 (17)	0.0480 (4)	
C4	0.6920 (2)	0.08023 (14)	0.47593 (18)	0.0484 (4)	
C7	0.8095 (2)	0.11507 (15)	0.1996 (2)	0.0549 (5)	
C3	0.6335 (2)	0.06052 (14)	0.6196 (2)	0.0536 (5)	
H3B	0.6757	0.0113	0.6774	0.064*	
C9	0.6309 (2)	0.14953 (15)	0.3821 (2)	0.0603 (5)	
H9A	0.5496	0.1851	0.4103	0.072*	
C5	0.8110 (2)	0.02762 (16)	0.4281 (2)	0.0612 (5)	
H5A	0.8527	−0.0204	0.4883	0.073*	
C6	0.8688 (2)	0.04527 (18)	0.2926 (2)	0.0656 (6)	
H6A	0.9493	0.0093	0.2636	0.079*	
C8	0.6893 (3)	0.16642 (16)	0.2469 (2)	0.0640 (6)	
H8A	0.6467	0.2137	0.1859	0.077*	
C10	0.8716 (3)	0.1343 (2)	0.0510 (2)	0.0766 (7)	
H10A	0.9542	0.0921	0.0370	0.115*	
H10B	0.9026	0.2012	0.0454	0.115*	
H10C	0.7976	0.1217	−0.0240	0.115*	
C1	0.1832 (3)	0.2597 (2)	0.8363 (3)	0.0953 (10)	
H1B	0.1537	0.3053	0.7611	0.143*	
H1C	0.2121	0.2946	0.9239	0.143*	
H1D	0.1026	0.2171	0.8564	0.143*	

### Atomic displacement parameters (Å^2^)

**Table:**

	*U*^11^	*U*^22^	*U*^33^	*U*^12^	*U*^13^	*U*^23^
S1	0.0813 (4)	0.0682 (4)	0.0421 (3)	0.0139 (3)	0.0186 (2)	0.00603 (19)
N3	0.0541 (10)	0.0515 (9)	0.0452 (8)	−0.0020 (7)	0.0137 (7)	0.0010 (6)
N2	0.0613 (11)	0.0587 (9)	0.0444 (8)	0.0068 (8)	0.0135 (7)	0.0048 (6)
N1	0.0608 (11)	0.0644 (10)	0.0491 (8)	0.0117 (8)	0.0193 (7)	0.0127 (7)
C2	0.0525 (11)	0.0490 (10)	0.0430 (9)	−0.0033 (8)	0.0081 (7)	−0.0011 (7)
C4	0.0456 (11)	0.0519 (10)	0.0481 (10)	−0.0013 (8)	0.0075 (7)	−0.0004 (7)
C7	0.0503 (12)	0.0649 (12)	0.0501 (10)	−0.0083 (9)	0.0095 (8)	0.0013 (8)
C3	0.0548 (12)	0.0547 (11)	0.0517 (10)	0.0023 (9)	0.0086 (8)	0.0040 (8)
C9	0.0606 (14)	0.0608 (13)	0.0605 (12)	0.0157 (10)	0.0146 (9)	0.0079 (9)
C5	0.0540 (13)	0.0715 (13)	0.0586 (11)	0.0135 (10)	0.0102 (8)	0.0107 (9)
C6	0.0510 (13)	0.0826 (15)	0.0644 (12)	0.0124 (10)	0.0180 (9)	0.0041 (10)
C8	0.0682 (15)	0.0639 (13)	0.0603 (11)	0.0094 (10)	0.0104 (9)	0.0147 (9)
C10	0.0778 (17)	0.0945 (17)	0.0587 (13)	−0.0095 (14)	0.0226 (11)	0.0069 (11)
C1	0.096 (2)	0.108 (2)	0.0849 (16)	0.0483 (16)	0.0451 (14)	0.0415 (14)

### Geometric parameters (Å, °)

**Table:**

S1—C2	1.6813 (17)	C3—H3B	0.9300
N3—C3	1.277 (2)	C9—C8	1.378 (3)
N3—N2	1.376 (2)	C9—H9A	0.9300
N2—C2	1.345 (2)	C5—C6	1.381 (3)
N2—H2A	0.8600	C5—H5A	0.9300
N1—C2	1.326 (2)	C6—H6A	0.9300
N1—C1	1.444 (3)	C8—H8A	0.9300
N1—H1A	0.8600	C10—H10A	0.9600
C4—C9	1.382 (3)	C10—H10B	0.9600
C4—C5	1.385 (3)	C10—H10C	0.9600
C4—C3	1.456 (2)	C1—H1B	0.9600
C7—C6	1.376 (3)	C1—H1C	0.9600
C7—C8	1.383 (3)	C1—H1D	0.9600
C7—C10	1.509 (2)		
			
C3—N3—N2	116.28 (16)	C6—C5—C4	121.15 (18)
C2—N2—N3	120.22 (16)	C6—C5—H5A	119.4
C2—N2—H2A	119.9	C4—C5—H5A	119.4
N3—N2—H2A	119.9	C7—C6—C5	121.32 (19)
C2—N1—C1	123.79 (16)	C7—C6—H6A	119.3
C2—N1—H1A	118.1	C5—C6—H6A	119.3
C1—N1—H1A	118.1	C9—C8—C7	121.85 (19)
N1—C2—N2	117.22 (15)	C9—C8—H8A	119.1
N1—C2—S1	123.41 (14)	C7—C8—H8A	119.1
N2—C2—S1	119.37 (14)	C7—C10—H10A	109.5
C9—C4—C5	117.71 (17)	C7—C10—H10B	109.5
C9—C4—C3	122.18 (17)	H10A—C10—H10B	109.5
C5—C4—C3	120.09 (17)	C7—C10—H10C	109.5
C6—C7—C8	117.33 (18)	H10A—C10—H10C	109.5
C6—C7—C10	121.6 (2)	H10B—C10—H10C	109.5
C8—C7—C10	121.1 (2)	N1—C1—H1B	109.5
N3—C3—C4	121.71 (18)	N1—C1—H1C	109.5
N3—C3—H3B	119.1	H1B—C1—H1C	109.5
C4—C3—H3B	119.1	N1—C1—H1D	109.5
C8—C9—C4	120.63 (18)	H1B—C1—H1D	109.5
C8—C9—H9A	119.7	H1C—C1—H1D	109.5
C4—C9—H9A	119.7		

### Hydrogen-bond geometry (Å, °)

**Table:**

*D*—H···*A*	*D*—H	H···*A*	*D*···*A*	*D*—H···*A*
N1—H1A···N3	0.86	2.27	2.644 (2)	107
N1—H1A···S1^i^	0.86	2.89	3.4869 (16)	128
N2—H2A···S1^ii^	0.86	2.57	3.4205 (18)	171

Symmetry codes: (i) *x*, −*y*+1/2, *z*−1/2; (ii) −*x*+1, −*y*, −*z*+2.
